# Analysis of Differentially Expressed Genes in a Chinese Cohort of Esophageal Squamous Cell Carcinoma

**DOI:** 10.7150/jca.40850

**Published:** 2020-04-06

**Authors:** Gang Liu, Yuan Zhao, Huili Chen, Jinru Jia, Xiaomin Cheng, Fengjie Wang, Qiang Ji, Rick F. Thorne, Song Chen, Xiaoying Liu

**Affiliations:** 1 Translational Research Institute, Henan Provincial People's Hospital, Academy of Medical Science, Zhengzhou University, Zhengzhou, 450003, China; 2 Biology Department, School of Life Sciences, Anhui Medical University, Hefei, 230032, China; 3 Department of Thoracic Surgery, the First Affiliated Hospital, Anhui Medical University, Hefei 230032, China; 4 Institute of Medicinal Biotechnology, Jiangsu College of Nursing, Huai'an, 223005, China

**Keywords:** ESCC, transcriptome, differentially expressed gene (DEG), RNA-seq, CLIC2, CLIC3, CLIC4

## Abstract

Esophageal squamous cell carcinoma (ESCC) is a leading malignancy in China with both high incidence and mortality. Towards improving outcomes, clinically-relevant biomarkers are urgently needed for use as prognostic and treatment targets. Herein we applied RNA-seq for deep sequencing of ten matched pairs of ESCC and adjacent non-cancerous tissues (NT) from Chinese patients. Transcriptomic data mapped to approximately 64% of all annotated genes with 2,047 and 708 unigenes being differentially up-regulated and down-regulated, respectively, between ESCCs and NT samples (*p*<0.05). Dividing cases by pathological grade revealed significant differentially expressed genes (DEG) between ESCC and NT in both low and high differentiation cases (*p*<0.05) whereas gene expression differences were not significantly different between high and low differentiation ESCC tissues (*p*=0.053). Moreover, the majority of ESCC and NT tissues formed clusters in principal component analyses. The veracity of the DEG list was validated in a larger cohort of 45 patient samples, with down-regulated CLIC3, up-regulated CLIC4 and unchanged expression of CLIC2 confirmed in ESCC using quantitative PCR and Western blotting. Our data reveal both previously identified ESCC biomarkers along with novel candidates and represent a ready resource of DEGs in ESCC for further investigation.

## Introduction

Esophageal cancer (EC) ranks as the sixth most common cancer worldwide and is the fourth most common cause of cancer deaths in China 1, 2. Nearly 500,000 new cases of EC are diagnosed every year and the often late discovery of this cancer is associated with poor prognosis with only a 5-year survival rate of ~14% 3, 4. Of the two histopathological types, esophageal adenocarcinoma (EADC) and esophageal squamous cell carcinoma (ESCC), the latter accounts for 80% of EC cases worldwide and it is the predominant form in Asia countries, exhibiting both high incidence and mortality 5, 6.

ESCCs are amongst the most aggressive tumors, and while rates in Europe and North America are comparatively low, the incidence of ESCC in Eastern Asia along with Eastern and Southern Africa is high and is increasing, although the reasons for this are not clear 4, 7, 8. The pathogenesis of ESCC still remains unclear with genetic and environmental factors thought to both contribute 4. The wide geographic and cultural variations in the incidence of ESCC emphasize the environmental association, for example, smoking and alcohol abuse appear as major contributors to ESCC burden in Western populations along with dietary habits, while in China, salty, hot and fumigated foods and drinks have been reported to contribute to high ESCC prevalence 9, 10. The epidemiological evidence is supported by animal studies which suggest that oxidative damage from factors such as smoking or gastroesophageal reflux can cause inflammation, esophagitis, and increased cell turnover, and thus initiate transformative events leading to ESCC 11. Nevertheless, the molecular mechanism(s) of ESCC onset and progression are still to be fully elucidated. A lack of specific biomarkers and effective therapeutic targets in ESCC are also major obstacles for improving the prognosis and extending the survival of patients.

With advances in the next-generation sequencing technologies, RNA sequencing (RNA-seq) has become a useful tool in defining whole cell transcriptomes 12. RNA-seq coupled with differentially expressed gene (DEG) profiling has been previously used to identify mRNA expression patterns in ESCC tissues 12-14 along with related analyses of microRNAs and long non-coding RNAs (lncRNA) 15-18. Herein, we reported the transcriptome of ESCCs and paired normal tissues (NTs) including high and low differentiation ESCC tissues from ten Chinese ESCC patients using deep sequencing. We compare the expression profiles of mRNA between ESCCs and NTs to identify DEGs that could contribute to the development and progression of ESCC. This analysis provided important clues for understanding the molecular mechanisms of ESCC pathogenesis and confirmed that differences were significant between ESCCs and NTs, however, differences were not significant between highly differentiated and low differentiated ESCC in Chinese patients. To validate our dataset, we analyzed the expression of members of the intracellular chloride ion channel (CLIC) family using real-time PCR (qPCR) and Western blotting. This analysis confirmed the predicted differential expression of CLIC mRNAs and proteins in ESCC. Thus our study provides further insights into the transcriptomes of Chinese ESCC patients and constitutes a verified resource for further molecular investigation of this disease.

## Materials and Methods

### Ethics

This study was approved by the institutional review board of Anhui Medical University (20170230). Written informed consent with a signature was obtained from each patient.

### Patient samples

A total of 45 paired fresh-frozen ESCC tissue samples and corresponding adjacent NTs were collected from 45 Chinese ESCC patients who underwent surgery at the First Affiliated Hospital of Anhui Medical University in 2016, China (Table 1). The patients include 21 women and 24 men. All tissues were collected from the mid- or lower esophagus and frozen in liquid nitrogen immediately after the operation and stored longer term at -80℃ until extraction of total RNA and protein. All tissues were independently validated by two professional pathologists using hematoxylin-eosin (HE) staining sections. The suffix ''a'' refers to the primary ESCC tissue and the ''b'' refers to the NT tissue.

### RNA-seq

Total RNA was extracted using the Total RNA Extractor (Trizol) Kit (Sangon Biotech Co., Ltd. Shanghai, China). The quality and quantity of total RNA were analyzed using an UltrasecTM 2100 pro UV/Visible Spectrophotometer (Amersham Biosciences, Uppsala, Sweden) and by agarose gel electrophoresis. The cDNA Synthesis Kit (Illumina Inc., San Diego, CA, USA) was used according to the manufacturer's recommendations to prepare cDNA for library construction and Illumina deep sequencing performed by Sangon Biotech Co., Ltd (Shanghai, China). Alignments were performed using the tool package SOAP2 developed for short oligo nucleotide analysis, allowing up to 2 mismatches with reference sequences. Sequenced reads were aligned to human transcript reference sequences from the ENSEMBL database (Homo_sapiens.GRCh37.55.cdna.all.fa) for expression analysis at gene/transcript levels.

### Real-time PCR

Total RNA was extracted from tissues using the Tissue RNA kit (OMEGA Bio Tek, USA) according to the manufacturer's instructions. The purity and concentration of RNA were determined according to 260/280 nm absorbance ratios using a NanoDrop 2000 spectrophotometer (ND-2000, Thermo Fisher Scientific, Waltham, MA, USA). One μg of total RNA was reverse transcribed using Novoscript® Plus All-in-one 1st Strand cDNA Synthesis Super Mix (gDNA Purge) (Novoprotein, China), qPCR analysis was performed using Novostart® SYBR qPCR Super Mix Plus Kit (Novoprotein, China) with a Quantstudio 6 Flex (Applied Biosystems, USA). For real-time PCR, 1 μl of the cDNA was used for mRNA amplification using KAPA SYBR FAST PCR Universal Kit (KapaBio systems) in a Mini-Opticon Thermal Cycler (BIORAD). Primers used for CLIC2, CLIC3 and CLIC4 with the specific forward and reverse primers designed in the 3' untranslated or other specific regions with Primer 5.0 software based on the assembled transcriptome sequences (Table 2).

### Western blotting

Tissues were homogenized using a Tissue Lyser LT (QIAGEN, Germany) in Cell Lysis Buffer (Beyotime, China). Protein concentrations were determined using the Modified Bradford Protein Assay Kit (Sangon Biotech, China). Thirty μg of total protein was resolved by SDS-PAGE, transferred to PVDF membrane (Bio-Rad, USA) and blocked for 1.5 h at RT in blocking solution (5 % skim milk w/v in Tris-Buffered Saline-Tween-20 (TBST)). Membrane was then incubated overnight at 4 ℃ with primary antibodies (CLIC2 ab-175230, Abcam, UK; CLIC3 15971-1-AP, GAPDH 60004-1-Ig, China Proteintech, China), with HRP-conjugated anti-Ig secondary antibodies (ZSbio, China) at RT for 1.5 h, and then with ECL-based detection solution. Densitometric analysis was performed using Image J normalization against the GAPDH loading control.

### Statistical analyses

Gene expression differences between and among groups were analyzed using the R vegan package (Version 2.0-2; Oksanen et al., 2011) in R v.2.8.1. Both *p* < 0.05 and *q* < 0.05 values were set as the threshold for determining DEGs. DEG identification and the significance of difference in gene expression analyses of each of the two groups was determined using the Dseq2 package (v1.12.4) in R v.2.8.1. Venn analysis used the VennDiagram package (VennDiagram_1.6.20.tar.gz) in R v.2.8.1. PCA analysis, Heatmap, Venn diagramvegan, TnnDiagram package (Version 2.0-2; Oksanen et al. 2011) in R v.2.8.1, respectively. Student's t and ANOVA test were carried out to determine the statistical significance between two and more groups, respectively (SPSS standard version 22.0, SPSS, Inc.). Statistical analyses of Western blotting performed with GraphPad Prism software Version 6.0 (San Diego, CA, USA). Correlation analyses between mRNA and protein expression were conducted by SPSS and Excel test. The criterion for statistical significance was considered *p*< 0.05.

Kaplan-Meier survival estimate was used to analyze the relationship between gene expression and ESCC patient survival using GEPIA data (http://gepia.cancer-pku.cn/detail.php).

## Results

### Transcriptomic analyses

Among the ESCC cohort, five highly differentiated and five low differentiated ESCC samples and their corresponding NTs were randomly selected for RNA-seq (Table 1). The highly differentiated samples were designated as Group A (n=5, Sample no.1a, 2a, 3a, 4a, 5a) and their NTs Group B (n=5, Sample no.1b, 2b, 3b, 4b and 5b). The low differentiation samples formed Group C (n=5, Sample no. 6a, 7a, 8a, 9a and 10a) and their NTs Group D (n=5, Sample no. 6b, 7b, 8b, 9b and 10b). The ten ESCC and paired NT tissues were then subjected to RNA-seq.

Average reads of at least 150 bp in length were obtained after removing adaptor sequences, low-quality and ambiguous “N” sequences providing a total of 975,307,954 high-quality reads and 1.46296×10^11^ bp, representing a large database of transcripts expressed in ESCCs and matched NTs. The numbers of clean reads and the assembled unigenes obtained through RPKM (reads per kilo bases per million mapped reads) are shown in Table 3. The mean clean read obtained was 46,241,913 with unigenes for individual samples mapping to about 64 % of all annotated genes. The read count ranged from 42,553,316 to 56,610,650, with the average total read count of 48,765,397. The total base count ranged from 63,82,997,400 to 8,491,597,500 bp with an average of 7.31×10^8^ bp. The average GC content was 52.24 %.

### Differentially expressed genes

**Figure 1:**
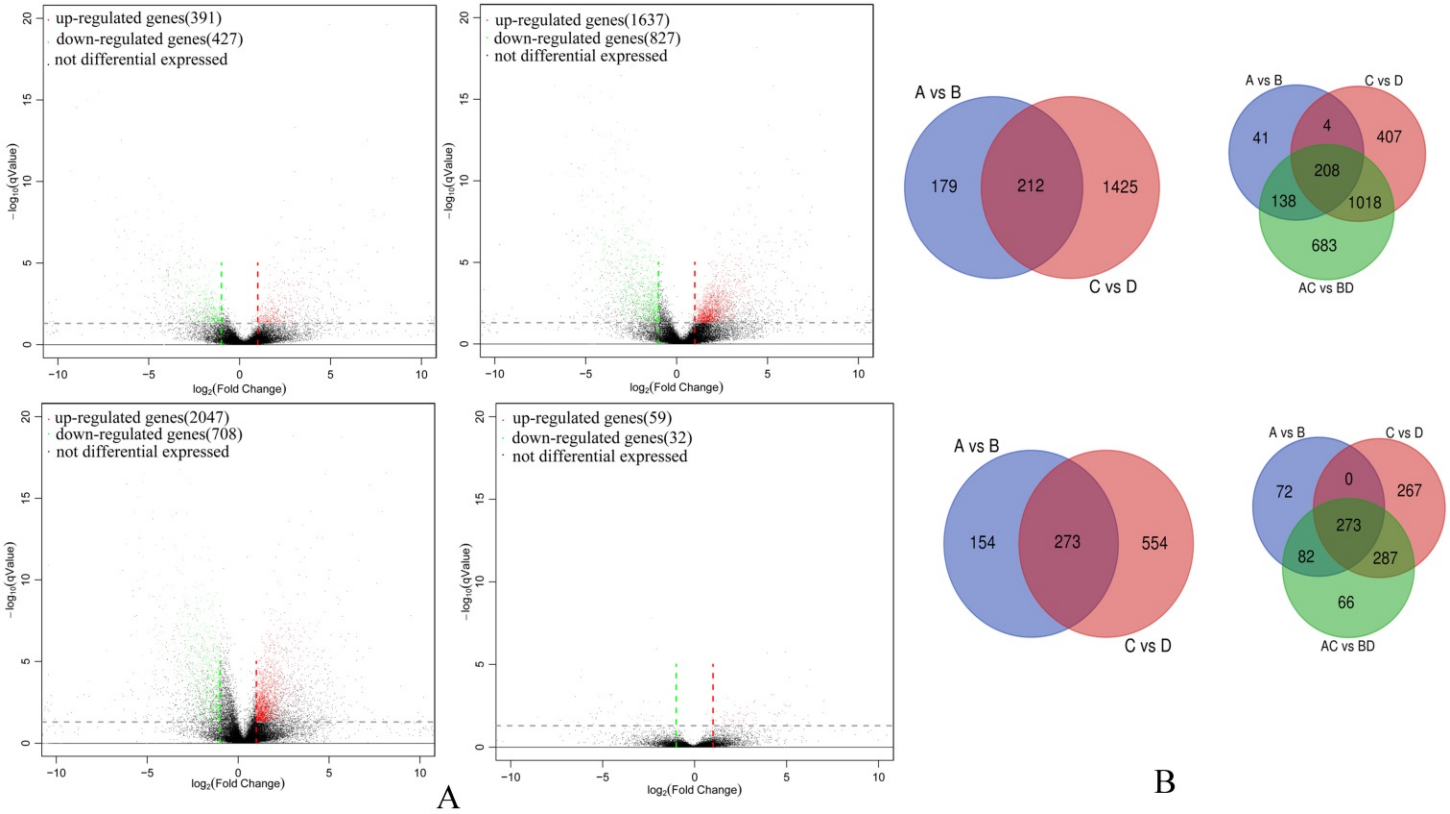
** Differentially expressed gene comparisons amongst ESCC cohort groups. (A)** Volcano plot of differentially expressed gene in Group A *vs* B (upper left), Group C *vs* D (upper right), Group A-C *vs* B-D (bottom right), and Group A *vs* C (bottom right). **(B)** Venn diagrams illustrating co-expressed up-regulated unigenes (upper) and co-expressed down-regulated unigenes between and among Groups (bottom).

A total of 6,128 unigenes were identified as differentially expressed between groups and among groups (Group A *vs* B, Group C *vs* D, Group A *vs* C, Group A-C *vs* B-D; Table S1). Amongst highly differentiated ESCCs there were 391 up-regulated and 427 down-regulated unigenes (Group A *vs* B) while the low differentiation samples in comparison had increased numbers of both up-regulated and down-regulated unigenes (Group C *vs* D, 1,637 and 827, respectively). Thus overall a total of 2,047 and 708 unigenes were up-regulated and down-regulated (Group A-C *vs* B-D), respectively. However, there were only 59 up-regulated and 32 down-regulated unigenes between highly differentiated ESCCs and low differentiated ESCCs (Group A *vs* C). In common between Group A *vs* B and Group C *vs* D there were 212 up-regulated and 273 down-regulated unigenes. Between and among group comparisons revealed 208 commonly up-regulated and 273 down-regulated unigenes. The differentially expressed gene profiles are depicted by Volcano plots (Figure 1A) with discrete and overlapping unigenes derived from each comparison (Group A *vs* B, Group C *vs* D, Group A-C *vs* B-D, and Group A *vs* C) shown using Venn diagrams (Figure 1B).

**Figure 2:**
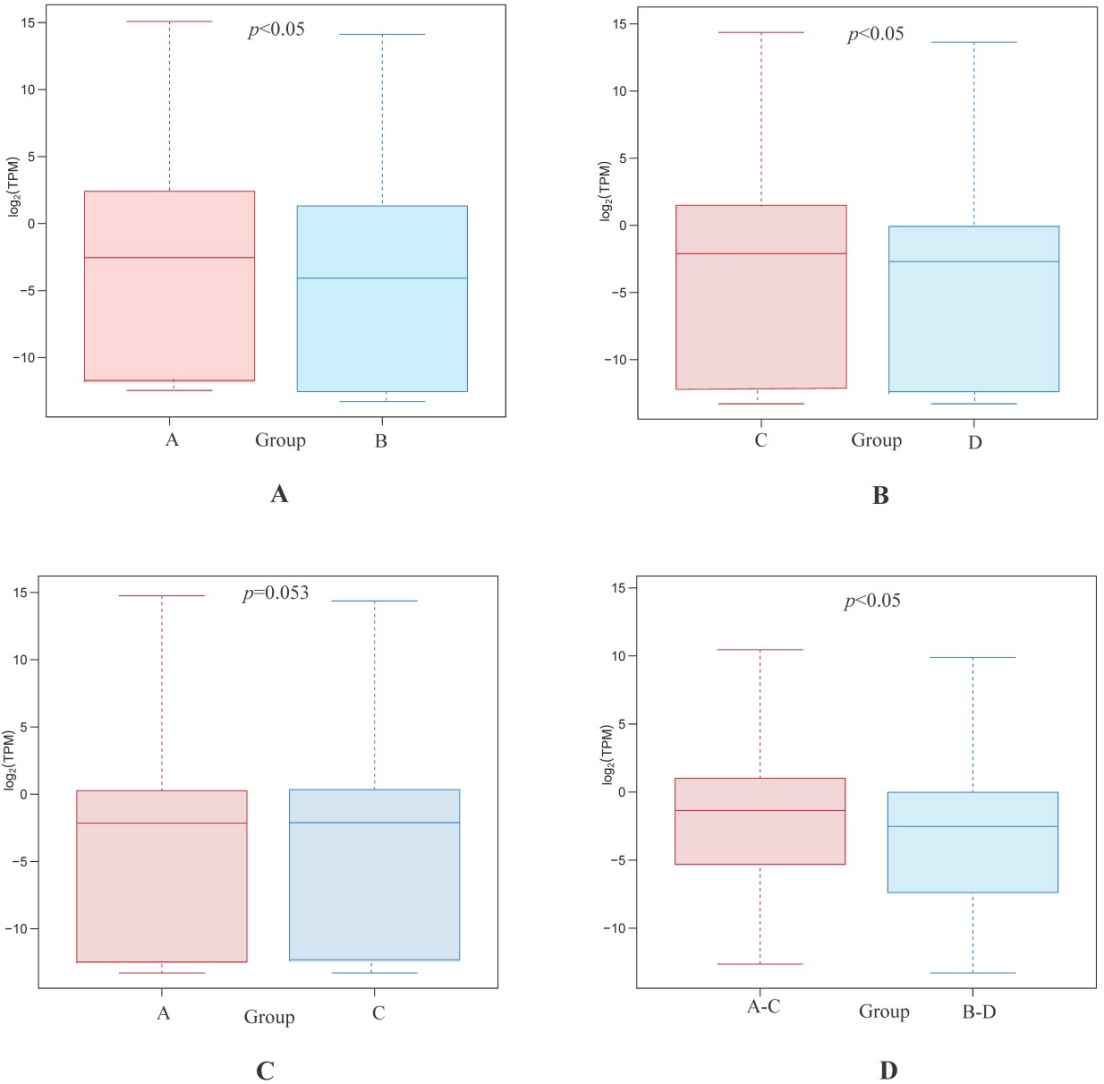
Differentially expressed gene between Group A and B (A), Group C and D (B), Group A and C (C), and Group A-C and B-D (D), respectively.

**Figure 3:**
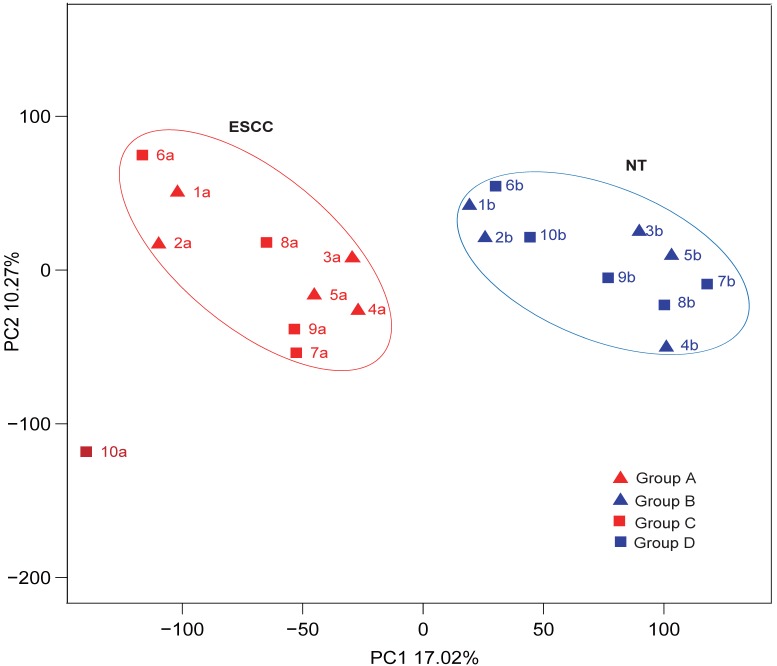
The characteristics of ESCCs and NTs from ten Chinese patients by PCA analysis.

Statistical analyses confirmed that gene expression differences were significant between both the highly differentiated ESCC tissues compared with paired NTs and the low differentiated ESCC tissues and paired NTs (*p*<0.05, Figures 2A and B, respectively). Interestingly, gene expression differences trended towards significance between high and low differentiation ESCC (Group A *vs* C, *p*=0.053 Figure 2C). Nevertheless, gene expression differences were significant between all ESCCs versus all NTs combined (Groups A-C* vs* B-D, *p*<0.05, Figure 2D). The overview of GO analysis results for the DEG with adjusted *p*-value is given in supplementary, and also indicated no significance between Group A and C (Figure S1). Principal component analysis (PCA) revealed clustering of all ESCCs with the exception of 10a, while all NTs separately clustered together (Figure 3). However, dissimilarities within ESCC samples in Group A appeared greater than in Group C, and similarly, dissimilarity within ESCC samples from Groups A-C was larger than in Groups B-D (Figure 3).

**Figure 4:**
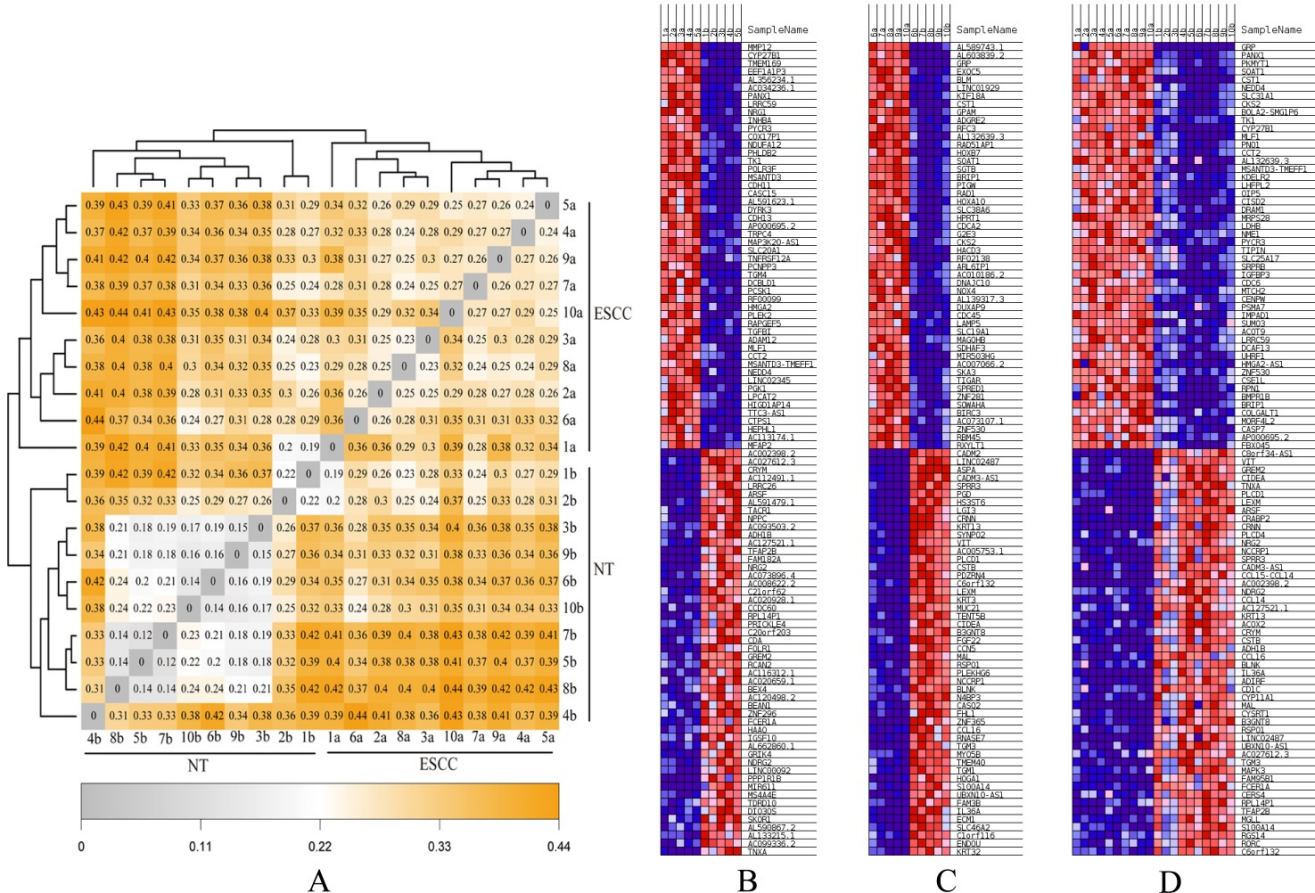
** The heatmaps for ESCCs and paired NTs from ten Chinese patients. (A) Genetic distance heatmap comparing ESCCs and NTs. (B-D) DEG heatmap comparisons between different groups of ESCCs and NTs.** (B) Group A *vs* B, (C) Group C *vs* D, and (D) Group A-C *vs* B-D comparisons of the top 50 DEGs.

Finally, hierarchical analyses of gene expression based on heatmap showed that all ESCC (Group A-C) and NTs (Group B-D) discretely clustered into two groups (Figures 4A, B). Individuals from Group A and Group C were irregularly distributed according to the ESCC clade, which was similar to NT clade (Group B and Group D). In addition, genetic distance was smaller in the two ESCC groups (Group A and C), but much greater between ESCC groups (Group A-C) and the NT Groups (Group B-D), consistent with the PCA analysis result (Figure 3). The heatmap results also indicated highly/low differentiation of ESCC tissues and paired NTs clustered together, respectively (Figure 4C, D).

### GSEA Analyses

**Figure 5:**
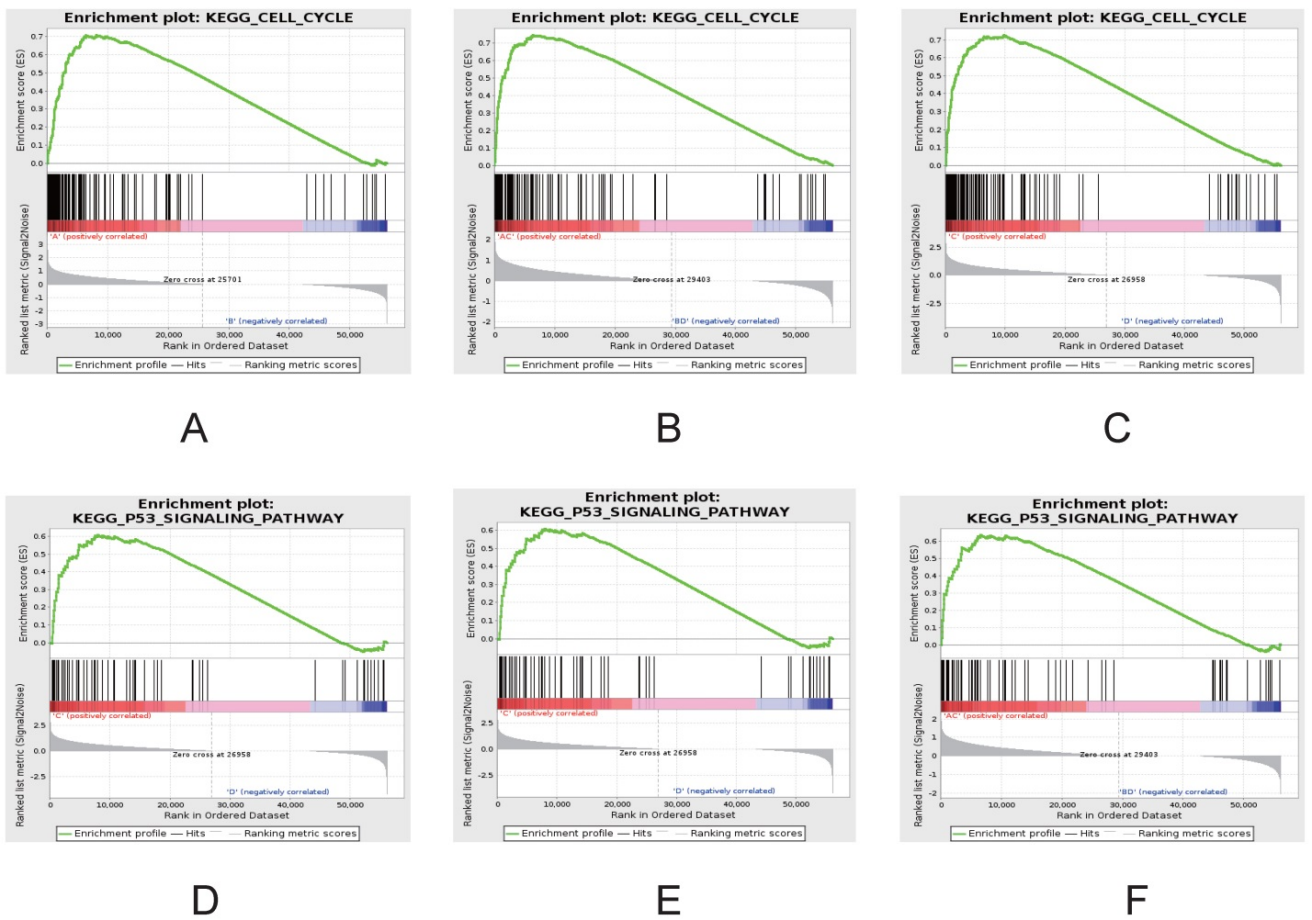
** GSEA demonstrating enhanced activity of cell cycle (A-C) and P53 signaling pathways (D-F) in ESCC.** Comparisons between groups A *vs* B (A, D), groups C *vs* D (B, E) and groups A-C *vs* B-D (C, F).

To further investigate the differences between ESCC and NTs, gene set enrichment analysis (GSEA) was used to investigate the specific gene expression patterns and pathways (Figure 5). The heatmap of GSEA between ESCC and NTs demonstrated showed that all ESCC and NTs discretely clustered into two groups. Further that gene sets also indicated that some gene sets and pathways were demonstrated enhanced activity. For example, GSEA results showed that cell cycle and P53 signaling pathways were significantly enriched in ESCC (Figure 5D-F).

### Verification of differentially expressed gene

In order to establish the veracity of our data as a resource for further study, it was necessary to perform secondary validation. Analysis of the differential expression of each of the six CLIC gene family members indicated that four of the CLIC genes (CLIC1, CLIC2, CLIC5 and CLIC6) were largely unchanged while CLIC3 was down-regulated and CLIC4 was up-regulated in ESCC, respectively (Table 4). Notably, while CLIC3 and CLIC4 were significantly changed they were not amongst the top 50 altered genes uncovered (see Figure 4B, C, D). For verification purposes we chose to analyze CLIC2, CLIC3 and CLIC4 expression in a larger cohort of ESCC samples.

**Figure 6:**
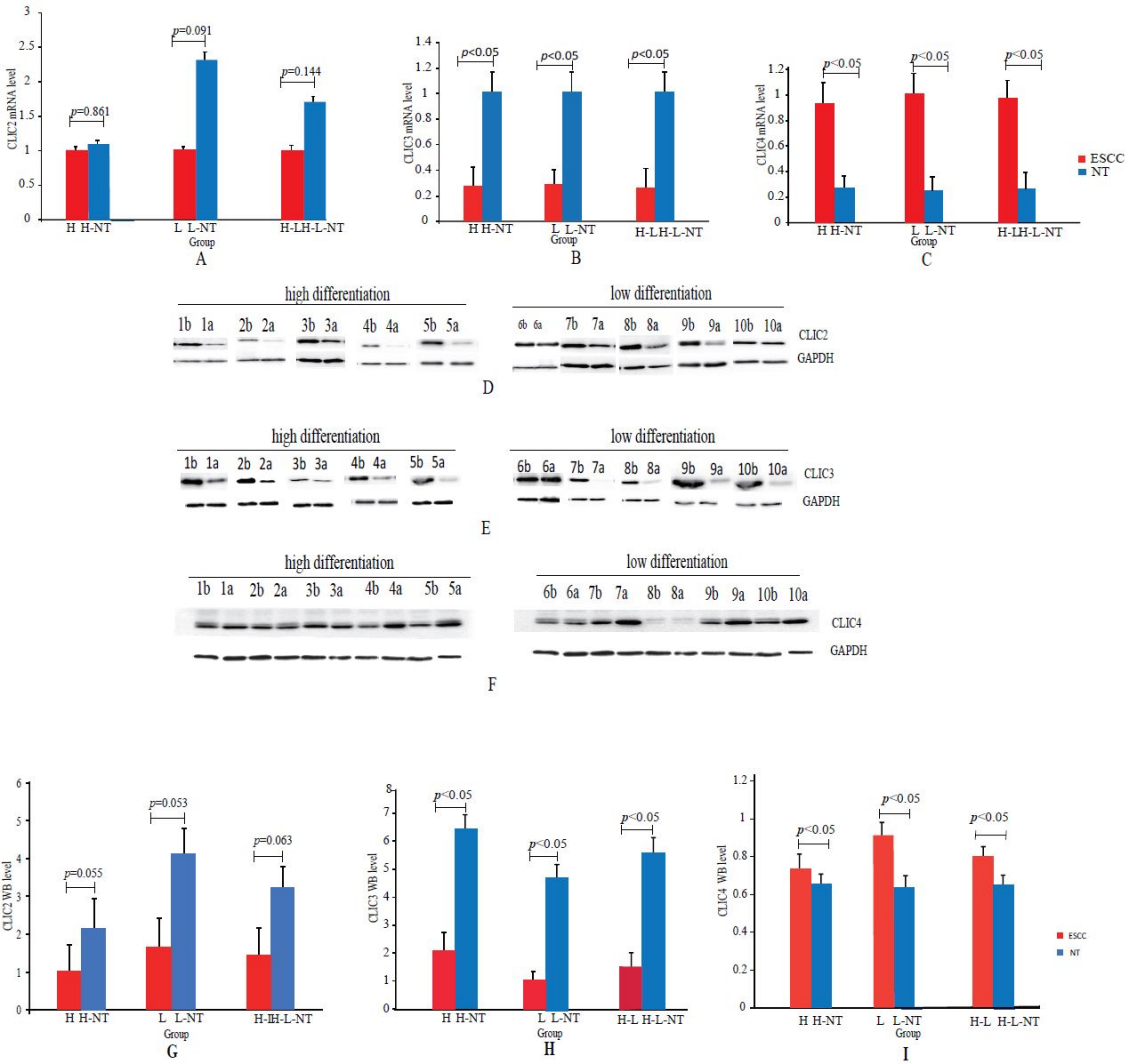
** Real-time PCR and Western blotting analyses of CLIC2, CLIC3 and CLIC4 in ESCCs and paired NTs. (A-C):** CLIC2 (A), CLIC3 (B) and CLIC4 (C) mRNA assays by qPCR between ESCCs and paired NTs.** (D-F):** Representative CLIC2 (D), CLIC3 (E) and CLIC4 (F) protein assays by Western blotting in ESCCs and paired NTs, GAPDH served as loading control. (**G-I):** Statistical analyses of CLIC2 (G), CLIC3 (H) and CLIC4 (I) protein levels between ESCCs and paired NTs.

We examined CLIC2, CLIC3 and CLIC4 mRNA and protein expression by qPCR and Western blotting analyses, respectively, in both ESCC and NT tissues collected from 45 patients. This cohort included 21 H (H = highly differentiated ESCCs and H-NT= paired NT) and 24 L (L = low differentiated ESCC and L-NT = paired NTs). Since there were no differences observed between DEGs in the H versus L groups, we combined all pathological grades for these analyses. QPCR assays indicated that CLIC2 mRNA was not significantly changed (*p*>0.05) between ESCC and NT groups (H *vs* H-NT, L *vs* L-NT and H-L *vs* H-L-NT) whereas CLIC3 and CLIC4 mRNA were down-regulated (*p*<0.05) and upregulated (*p*<0.05) in ESCCs, respectively (Figures 6A-C).

**Figure 7:**
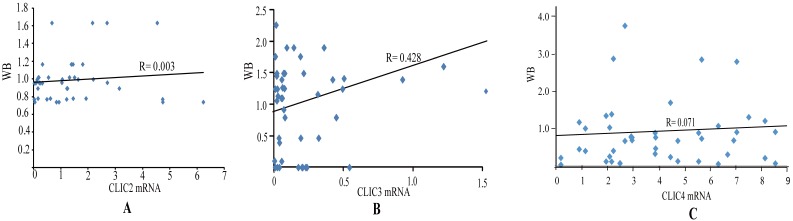
** Correlation analysis of mRNA and protein level of CLIC2, CLIC3 and CLIC4 in ESCC tissues.** Pearson correlation between mRNA and protein level of CLIC2 (n=45) (A), CLIC3 (n=45) (B), CLIC4 (n=45) (C).

**Figure 8:**
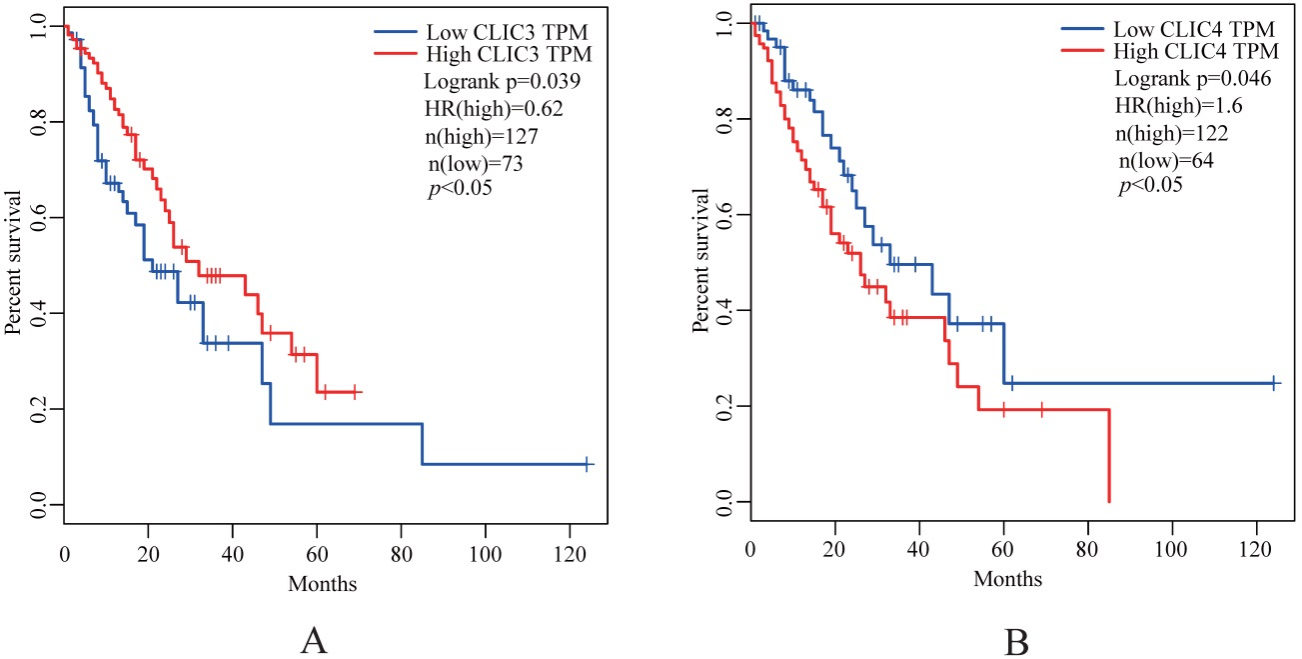
Kaplan-Meier analysis of the probability of overall survival (OS) of ESCC patients (N=180) derived from the GEPIA. The optimal custom cut-off points were used for CLIC3 (A) and CLIC4 (B), respectively.

The expression of CLIC2, CLIC3 and CLIC4 protein similarly demonstrated that CLIC2 protein levels were not altered amongst ESSC versus NT groups while CLIC3 and CLIC4 protein expression reflected the changes observed in mRNA analyses (Figure 6D-I). Bivariate comparisons between mRNA and protein revealed significant Pearson correlation coefficients for CLIC3 and CLIC4 (R=0.428 and R=0.051, respectively) while no significant correlation was observed between mRNA and protein levels of CLIC2 (n=45, R=0.003) (Figure 7). Thus the expression differences in CLIC genes predicted from the RNA-seq data appear maintained in independent comparisons in a larger cohort of ESCC samples. Of note, low level of CLIC3 and high level of CLIC4 expression appeared to be associated with poor overall survival (OS) of ESCC patients included in the GEPIA dataset (Figure 8A-B). Nevertheless, no significant relationship was found between CLIC2 expression levels and patient prognosis.

## Discussion

Deep sequencing using RNA-seq has been widely applied to help understand the underlying basis of disease, especially cancer and the identification of novel tumor biomarkers represents a large part of all dedicated research efforts 14. From the most reliable transcriptomic data using about 2.5-3 million reads, about 15,000 genes can be detected inferring that about 50% of all genes in the human genome are expressed in any given tissue 12, 19, 20. In this study, we have compared the gene expression profiles between differently ESCCs and paired NTs used RNA-seq. These data revealed both previously identified ESCC biomarkers along with novel candidates and represent a ready resource of DEGs in ESCC for further investigation.

Our analysis of transcriptomic data was centered on comparing paired ESCC versus NT samples with additional consideration of ESCC differentiation status. In the clinical setting, ESCC cases presenting with low pathological differentiation have general worse prognoses than high differentiation cases 2. Overall we found that transcriptomic profiles between ESCCs and NTs were dramatically changed, consistent with prior studies 21-23. Moreover, there were substantially more up-regulated unigenes in ESCC versus NT comparisons compared to the number of down-regulated unigenes. As might be anticipated, the numbers of differentially expressed genes between normal and tumor samples were comparably increased in low versus high differentiation cases. However, gene expression profiles between high and low differentiation tumors failed to reach statistical significance.

Examination of these data by PCA analysis revealed that ESCC and NT tissues clustered together, but nevertheless, the intra-cluster variation appeared broad. Since close proximity in PCA plots is a measure of sameness, the dispersed locations of high and low differentiation cases points to limited predictive ability of pathology grade to define gene expression profiles. Indeed a recent publication that described four molecular subtypes of ESCC found that those designated ESCC2 were more likely to be poorly differentiated and metastatic compared with well-differentiated ESCC1 cases 24. Nevertheless, another distinct classifier called ESCC4 that is also associated with worse survival has distinct changes including chromosomal instability and a high frequency of loss of heterozygosity. Whether or not these molecular classifications will stand-up in the long term remains to be determined since there is remarkable molecular heterogeneity in ESCCs both within and between geographical populations 10.

Amongst the differentially expressed ESCC genes identified, some hits have been highlighted in previous studies, including MCM4, MMP1, MMP12, SLC22A3 along with members of the CLIC family 25-27. Furthermore, based on the results of the GSEA analysis, p53 pathways along with cell cycle regulation were also de-regulated in ESCC, a finding that had been reported previously 28. For validating our datasets we chose the CLIC family of genes, not because they represented the most highly over- or under-expressed genes in ESCC. Rather, we elected to verify selected CLIC expression (representative unchanged, up-regulated and down-regulated genes) in a larger cohort of samples since this analysis would provide a broader indication of the reliability of the overall gene lists. Indeed the differential expression profiles predicted for each CLIC gene from the RNA-seq training set were subsequently confirmed in the larger validation cohort. Nevertheless, comparing mRNA expression versus protein on a case-by-case basis highlights that high mRNA levels did not always reflect high protein expression and vice versa, especially in tumors 29, 30. This may of course reflect the biology or technical problems in dealing with clinical samples which can be inherently variable even when systematically collected.

From the biological perspective, the chloride intracellular channel (CLIC) family represented by CLIC1-6 constitutes a subgroup of the glutathione-S- transferase (GST) superfamily 31-33. Ion channels play important roles in the development of cancer and their expression is known to be altered in cancer cells 12, 34. Chloride channel proteins are ubiquitously expressed, and the development and progression of some cancers has been proposed to be associated with the up-regulation of CLIC1, CLIC3 and 4 34, 35. Although CLIC2 was shown to be significant expressed in non-cancer tissues in studies of human hepatocellular and metastatic colorectal carcinomas 33, we found CLIC2 not to be differentially expressed in ESCC versus NT groups. However, our combined analysis by RNA-seq, qPCR and Western blotting indicated that CLIC3 was significantly down-regulated in ESSC tissues. Previous studies have shown that, the gene expression of CLIC3 was significantly increased compared to healthy controls in human cancer, such as malignant pleura mesothelioma (MPM) 35-37. While our findings concerning CLIC3 in ESCC are different to that observed in MPM, suggests that further functional investigations of the role of CLIC3 in ESCC and other cancers may be warranted. We found CLIC4 was significantly differentially expressed in ESCC and paired NTs based on RNA-seq, qPCR and Western blotting, which were similar to previous studies 34. Moreover, our results showed that low level of CLIC3 and high level of CLIC4 were associated with poor OS of ESCC patients, suggesting that further investigations on the potential of these genes as prognostic biomarkers in ESCC patients are warranted 38, 39.

In summary, we conducted comprehensive transcriptome sequencing of ESCC and paired NTs to derive a substantial list of de-regulated genes. Up-regulated and down-regulated genes were identified, suggesting the power and sensitivity of an RNA-seq based approach. We propose this data provides important clues for understanding the molecular mechanisms of ESCC pathogenesis 4, 10, 14.

## Supplementary Material

Supplementary figure.

Supplementary table.

## Figures and Tables

**Table 1:** The clinicopathological characteristics of the ten cases of Chinese ESCC.

Patient ID	Case no.	Sex	Tumor size (cm)	Treatment	Histology type	differentiation	Primary location	Family history of cancer	Adjuvant Chemotherapy
1	2016018801	Male	2.8×2.0×1.0	Resection	ESCC	high	Middle esophagus	Negative	None
2	2016019732	Male	2.8×2.0×1.0	Resection	ESCC	high	Low esophagus	Negative	None
3	2016019942	Male	5.0×3.0×2.0	Resection	ESCC	high	Low esophagus	Negative	None
4	2016028925	Male	5.0×3.5×0.6	Resection	ESCC	high	Middle esophagus	Negative	None
5	2016035209	Female	4.0×2.1×1.1	Resection	ESCC	high	Middle esophagus	Negative	None
6	2016018228	Female	2.0×1.2×0.7	Resection	ESCC	low	Middle esophagus	Negative	None
7	2016027858	Male	3.7×3.3×1.3	Resection	ESCC	low	Middle esophagus	Negative	None
8	2016035368	Male	3.0×3.0×1.0	Resection	ESCC	low	Middle esophagus	Negative	None
9	2016037531	Male	6.5×3.0×1.1	Resection	ESCC	low	Middle esophagus	Negative	None
10	2016038506	Male	6.0×4.0×1.1	Resection	ESCC	low	Middle esophagus	Negative	None

**Table 2:** Primers of CLIC2, CLC3 and CLIC4 used in this study.

Gene name	Primer sequence (5'-3')
CLIC2	F: 5'-GACCCTGAGATTGAGCTTTTTG-3' R: 5'-AACTCCTTTAAGCCAGAGGATC-3'
CLC3	F: 5' -CAGATCGAGGACTTTCTGGAG-3' R: 5'-GGAGAACTTGTGGAAAACGTC-3'
CLIC4	F: 5'-ATGACATTAGCTGATTGCAACC-3' R: 5'-CGTCCCTACTGTATGCATTAGT-3'
GAPDH	F: 5'-CACCCACTCCTCCACCTTTG-3' R: 5'-CCACCACCCTGTTGCTGTAG-3'

**Table 3:** Summary of assembly and annotation results for ESCCs and NTs from ten Chinese patients in this study.

Group	Sample no.	Reads count	Bases count (bp)	GC bases count (bp)	GC (%)
A	1a	44,849,202	6,727,380,300	3,461,726,497	51.46%
	2a	48,160,630	7,224,094,500	3,675,204,465	50.87%
	3a	51,676,610	7,751,491,500	3,935,742,195	50.77%
	4a	5,1336,588	7,700,488,200	4,011,882,197	52.10%
	5a	49,105,978	7,365,896,700	3,719,188,635	50.49%
B	1b	54,150,646	8,122,596,900	4,114,541,533	50.66%
	2b	48,994,144	7,349,121,600	3,731,856,656	50.78%
	3b	48,813,576	7,322,036,400	3,937,277,257	53.77%
	4b	45,264,310	6,789,646,500	3,945,468,046	58.11%
	5b	48,458,666	7,268,799,900	4,036,845,872	55.54%
C	6a	50,065,412	7,509,811,800	3,684,072,907	49.06%
	7a	50,204,384	7,530,657,600	3,757,083,386	49.89%
	8a	51,844,356	7,776,653,400	3,916,995,824	50.37%
	9a	44,370,636	6,655,595,400	3,376,536,600	50.73%
	10a	42,553,316	6,382,997,400	3,274,611,235	51.30%
D	6b	51,768,224	7,765,233,600	4,032,495,670	51.93%
	7b	50,776,086	7,616,412,900	4,252,296,568	55.83%
	8b	43,064,778	6,459,716,700	3,634,881,053	56.27%
	9b	43,239,762	6,485,964,300	3,454,177,107	53.26%
	10b	56,610,650	8,49,1597,500	4,379,400,449	51.57%

**Table 4:** Differential expression of CLIC1-6 in this study.

Comparison	CLIC member					
	CLIC1	CLIC2	CLIC3	CLIC4	CLIC5	CLIC6
A *vs* B^ a^	0.2256	0.3452	-2.5706^***^	1.1859	0.0837	0.7177
C *vs* D^ a^	0.1563	1.5381^**^	-2.6464^***^	1.6611^**^	-0.0091	1.3687
AC *vs* BD^ a^	0.1893	0.8969	-2.6171^***^	1.3788^**^	0.0445	1.0738

(a) log_2_Fold change; (*) *p*<0.05, (**) *p*<0.01, (***) *p*<0.001
