# Supplementary figures and images for: Analysis of Differentially Expressed Genes in a Chinese Cohort of Esophageal Squamous Cell Carcinoma
Source: J Cancer. 2020 Apr 6;11(13):3783–93. doi: 10.7150/jca.40850 (PMC7171491; doi:10.7150/jca.40850)

- 1 **Figure S1. GO analysis results for the differentially expressed gene between Group A and C.**
- 2

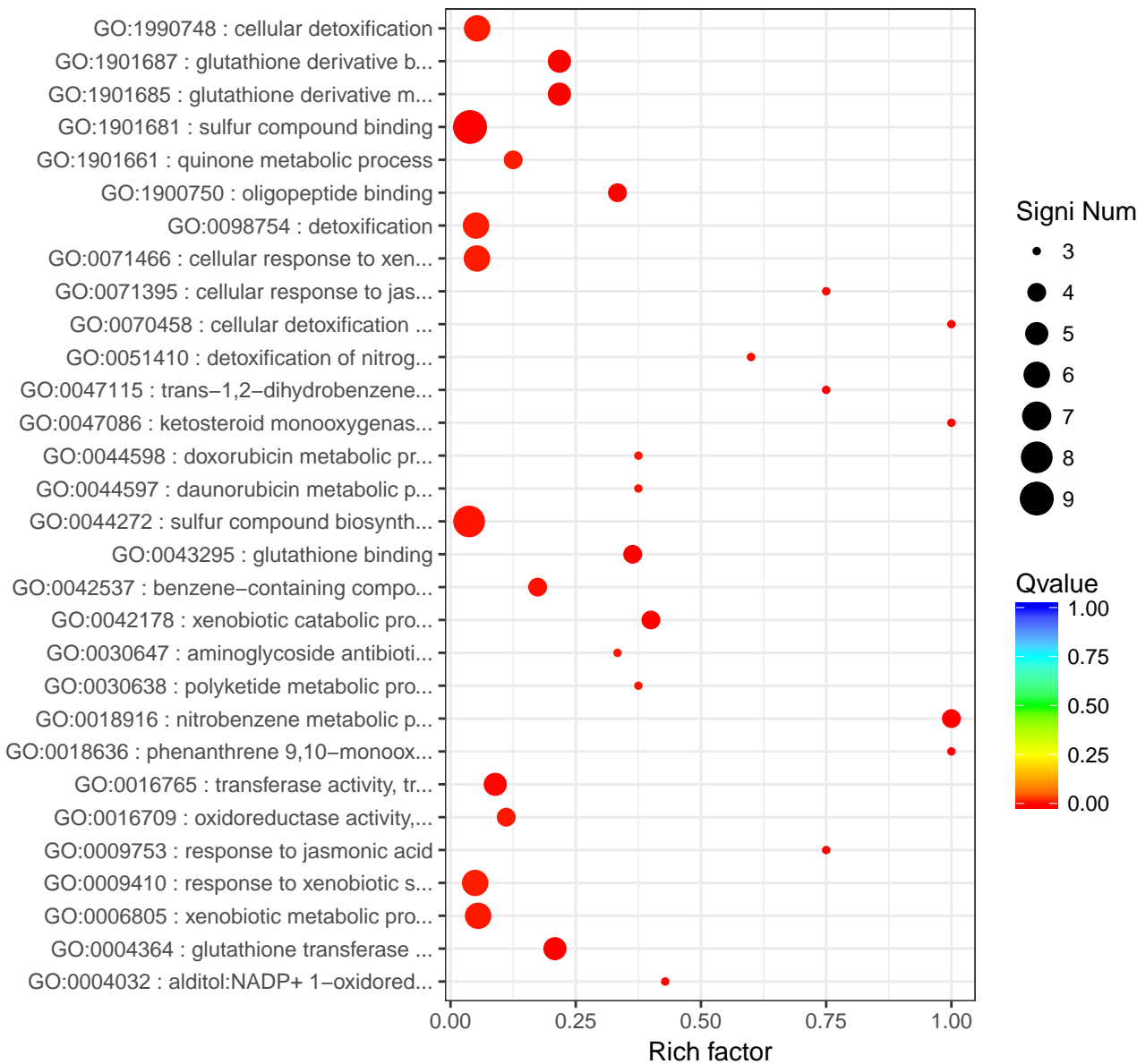

Supplement: Supplementary file 1 — Supplementary figure. [file jcav11p3783s1.pdf]
